# Short-Term Variations in Neutrophil-to-Lymphocyte and Urea-to-Creatinine Ratios Anticipate Intensive Care Unit Admission of COVID-19 Patients in the Emergency Department

**DOI:** 10.3389/fmed.2020.625176

**Published:** 2021-01-20

**Authors:** Antonio Giovanni Solimando, Nicola Susca, Paola Borrelli, Marcella Prete, Gianfranco Lauletta, Fabrizio Pappagallo, Roberta Buono, Gianfranco Inglese, Bianca Maria Forina, Donatello Bochicchio, Martina Capobianco, Valeria Carrieri, Sebastiano Cicco, Patrizia Leone, Nicola Silvestris, Annalisa Saracino, Roberto Ria, Vito Procacci, Giovanni Migliore, Angelo Vacca, Vito Racanelli

**Affiliations:** ^1^Guido Baccelli Unit of Internal Medicine, Department of Biomedical Sciences and Human Oncology, School of Medicine, Aldo Moro University of Bari, Bari, Italy; ^2^Medical Oncology Unit, IRCCS Istituto Tumori “Giovanni Paolo II” of Bari, Bari, Italy; ^3^Laboratory of Biostatistics, Department of Medical, Oral and Biotechnological Sciences, G. d'Annunzio University of Chieti-Pescara, Chieti, Italy; ^4^Department of Biomedical Sciences and Human Oncology, School of Medicine, Aldo Moro University of Bari, Bari, Italy; ^5^Infectious Diseases Unit, Department of Biomedical Sciences and Human Oncology, School of Medicine, Aldo Moro University of Bari, Bari, Italy; ^6^Emergency Department, Azienda Ospedaliero-Universitaria Policlinico, Bari, Italy; ^7^Azienda Ospedaliero-Universitaria Policlinico, Bari, Italy

**Keywords:** COVID-19, disease severity, intensive care unit, neutrophil-to-lymphocyte ratio, urea-to-creatinine ratio

## Abstract

**Background:** Timely assessment of COVID-19 severity is crucial for the rapid provision of appropriate treatments. Definitive criteria for the early identification of severe COVID-19 cases that require intensive care unit admission are lacking.

**Methods:** This was a single-center, retrospective case-control study of 95 consecutive adults admitted to the intensive care unit (cases) or a medical ward (controls) for laboratory-confirmed COVID-19. Clinical data were collected and changes in laboratory test results were calculated between presentation at the emergency department and admission. Univariate and multivariable logistic regression was performed to calculate odds ratios for intensive care unit admission according to changes in laboratory variables.

**Results:** Of the 95 adults with COVID-19, 25 were admitted to intensive care and 70 to a medical ward after a median 6 h stay in the emergency department. During this interval, neutrophil counts increased in cases and decreased in controls (median, 934 vs. −295 × 10^6^/L; *P* = 0.006), while lymphocyte counts decreased in cases and increased in controls (median, −184 vs. 109 × 10^6^/L; *P* < 0.001). In cases, the neutrophil-to-lymphocyte ratio increased 6-fold and the urea-to-creatinine ratio increased 20-fold during the emergency department stay, but these ratios did not change in controls (*P* < 0.001 for both comparisons). By multivariable logistic regression, short-term increases in the neutrophil-to-lymphocyte ratio (OR = 1.43; 95% CI, 1.16–1.76) and urea-to-creatinine ratio (OR = 1.72; 95% CI, 1.20–2.66) were independent predictors of intensive care unit admission.

**Conclusion:** Short-time changes in neutrophil-to-lymphocyte ratio and urea-to-creatinine ratio emerged as stand-alone parameters able to identify patients with aggressive disease at an early stage.

## Introduction

The first case of coronavirus disease 2019 (COVID-19) in Italy was reported on February 20, 2020. Shortly after, the number of cases registered throughout the country exceeded those in China (1), and today Italy ranks as one of the most affected countries (2) in terms of both total cases per 10,000 population and case-fatality rate (https://covid19.who.int/). The clinical spectrum of COVID-19 appears to be wide, including asymptomatic infection, mild upper respiratory tract illness, and severe pneumonia with respiratory failure and even death (3–6). Even patients with initially mild illness without radiographic abnormalities may suddenly worsen and require intensive care (1, 7–9), making it challenging to estimate disease severity in the early phase. Patients who present to the emergency department with suspected COVID-19 need to be monitored, to anticipate clinical deterioration and initiate appropriate clinical management and treatments as needed.

The course of infection with the virus that causes COVID-19, namely severe acute respiratory syndrome coronavirus 2 (SARS-CoV-2), is just beginning to be studied, and early indicators of severe disease are lacking. An accurate model of disease progression would help physicians assess each patient's risks and choose the required intensity of care at the moment of admission. So far, various prognostic factors have been identified (10, 11), but all of them are static indicators that provide only snapshots of a process that is rapidly evolving (7). We hypothesized that dynamic observations of early changes in clinical variables can predict clinical deterioration requiring intensive care. This study therefore investigated the ability of rapid changes on a panel of clinical variables, measured in COVID-19 patients in the emergency department, to discriminate between those who require admission to an intensive care unit (ICU) and those who can be treated in a nonintensive medical ward.

## Methods

This retrospective case-control study was conducted at the Bari Policlinic, the largest academic hospital in Puglia Region. The study protocol was approved by the institutional ethics board, which waived the need for informed consent, and was performed in accordance with the ethical standards laid down in the 1964 Declaration of Helsinki and its later amendments.

The study considered all adults (aged ≥18 years) who presented to the Emergency Department between March 13 and April 1, 2020 (during the peak of the outbreak in Puglia Region) with suspected COVID-19, and were subsequently admitted to either the ICU (cases) or a medical ward (non-ICU, controls) for laboratory-confirmed SARS-CoV-2 infection.

Patients were identified from Edotto, the regional health information system. Clinical data, collected from Galileo electronic health records, included age, sex, smoking habit, triage vital signs and presenting symptoms, comorbidities, current medications, laboratory test results (pre-defined disease-specific panel), and duration and outcome of follow-up. Laboratory confirmation of SARS-CoV-2 was defined by a positive result on a real-time reverse transcriptase-polymerase chain reaction (RT-PCR) assay performed on nasopharyngeal swabs or lower respiratory tract aspirates.

### Statistical Analyses

Qualitative variables were described in terms of absolute numbers and percentages. Associations between qualitative variables and group assignment were tested for significance using Pearson's chi square or Fisher's exact test. Quantitative variables were described using median and interquartile range (IQR). The Shapiro-Wilk test indicated that quantitative data were not normally distributed, so the non-parametric Wilcoxon rank-sum test was used to compare these variables (and their changes over time) between case and control groups.

Survival analysis was performed by applying the Kaplan-Meier estimator and log-rank test for equality of survivor functions. The association between clinical variables and overall survival was analyzed with the Cox model of proportional hazards, and the assumption was verified by the Schoenfeld test.

Correlations among selected variables at baseline (t^0^) were tested using Spearman's rho, for cases and controls separately. These analyses were done only for variables with well-established pathophysiological connections and documented alterations in patients with MERS-CoV and SARS-CoV infection (12, 13), and they served to identify any variables that were associated with higher probability than the expected by chance.

Unadjusted odds ratios (crude ORs) and corresponding 95% CIs were calculated in univariate analyses using the Wald test to assess relationships between study group assignment (ICU vs. non-ICU) and changes in laboratory variables (Δ, t^1^-t^0^). Multivariable logistic regression was done to identify the mutually adjusted effect on group assignment of laboratory variables included on the basis of statistical significance in the univariate analyses, clinical judgment, and contribution to the model (likelihood-ratio test). Age and sex were used as adjusting variables. The goodness of fit of the regression model was assessed using the Hosmer-Lemeshow test. For all analyses, a *P*-value ≤ 0.05 was considered significant (two-sided). All analyses were performed using Stata software v15.1 (StataCorp, College Station, USA).

## Results

Of the 95 patients who presented to the emergency department for COVID-19 during the 20-day study period, 25 were admitted to the ICU (cases) and 70 were admitted to a non-ICU medical ward (controls). The patients underwent a medical examination and blood testing at presentation to the emergency department (t^0^) and had another blood panel done just prior to being transferred to an inpatient unit (t^1^). The median length of stay in the emergency department was 6 h (IQR, 3–19 h), during which time patients were observed and received initial treatments. Cases and controls were similar in terms of age and sex, with both groups having a predominance of men (Table 1). At presentation (t^0^), cases required the administration of higher fractions of oxygen (*P* = 0.01). Fever was the prevailing symptom in both groups, but cases more frequently were dyspneic (*P* = 0.01) and needed noninvasive ventilation (*P* < 0.001). Hypertension was the most common comorbidity in both groups, while obesity was seen only in cases (*P* < 0.001). Systemic corticosteroids were administered to one case (4.0%) and two controls (2.9%; *P* > 0.99), and intravenous fluids were used liberally in all patients.

**Table 1:** Clinical characteristics of cases and controls at presentation (t^0^).

**Variable**	**ICU (*n* = 25)**	**Non-ICU (*n* = 70)**	***P***
Age, median (IQR), y	71.0 (60.0–75.0)	60.0 (52.0–74.8)	0.19^a^
Sex, no. (%)			0.65^b^
Male	18 (72.0)	45 (64.3)	
Female	7 (28.0)	25 (35.7)	
Current smokers	0 (0)	2 (3.2)	>0.99^c^
Systolic arterial pressure, median (IQR), mmHg	127.0 (120.0–135.0)	125.0 (116.5, 140.0)	0.63^a^
Diastolic arterial pressure, median (IQR), mmHg	70.0 (67.5–78.5)	74.0 (70.0–80.0)	0.55^a^
Heart rate, median (IQR), bpm	93.5 (76.0–100.0)	90.0 (76.0–101.0)	0.71^a^
Body temperature, median (IQR), °C	36.7 (36.4–37.8)	37.0 (36.2–38.0)	0.81^a^
Peripheral capillary oxygen saturation, median (IQR), %	94.0 (92.5–98.0)	97.0 (94.3–98.0)	0.23^a^
Fraction of inspired oxygen, median (IQR), %	22.5 (21.0–75.0)	21.0 (21.0–21.0)	0.01^a^
**COVID-19 SYMPTOMS, NO. (%)**			
Fever history	23 (100.0)	59 (88.1)	0.11^c^
Dyspnea	19 (79.2)	31 (47.0)	0.01^b^
Noninvasive ventilation in ED	9 (45.0)	3 (4.9)	<0.001^b^
**COMORBIDITIES, NO. (%)**			
Hypertension	14 (60.9)	29 (51.8)	0.63^b^
Obesity	9 (37.5)	0 (0)	<0.001^c^
Chronic heart failure	4 (16.7)	9 (14.5)	0.75^c^
COPD	4 (16.7)	6 (9.7)	0.46^c^
Type 2 diabetes mellitus	3 (12.5)	12 (19.4)	0.54^c^
Allergy	3 (12.5)	5 (7.9)	0.68^c^
CNS disease	1 (4.2)	9 (14.5)	0.27^c^
Atrial fibrillation	1 (4.2)	5 (8.1)	>0.99^c^
Cancer	1 (4.2)	4 (6.5)	>0.99^c^
Chronic kidney disease (≥III KDOQI)	1 (4.2)	5 (8.1)	>0.99^c^
Autoimmune disease	1 (4.2)	1 (1.6)	0.48^c^
**CURRENT MEDICATIONS, NO. (%)**			
Antiplatelet drugs	6 (35.3)	9 (17.3)	0.17^c^
ACE inhibitors or ARB	4 (33.3)	14 (28.6)	0.74^c^
Other antihypertensive drugs	4 (33.3)	18 (36.7)	>0.99^c^
Inhalants	2 (11.8)	0 (0)	0.06^c^
Warfarin or DOAC	1 (5.9)	5 (9.4)	>0.99^c^
Systemic corticosteroids	0 (0)	2 (3.8)	>0.99^c^
Immunosuppressants	0 (0)	2 (3.8)	>0.99^c^
Low-molecular-weight heparin	0 (0)	0 (0.0)	>0.99^c^
Chemotherapy	0 (0)	2 (3.8)	>0.99^c^

a Wilcoxon's rank sum test;

b Pearson's chi-square test;

c *Fisher's exact test*.

*ACE, angiotensin-converting enzyme; ARB, angiotensin receptor blockers; CNS, central nervous system; COPD, chronic obstructive pulmonary disease; DOAC, direct oral anticoagulants; ED, emergency department; ICU, intensive care unit; KDOQI, Kidney Disease Outcomes Quality Initiative*.

The patients were followed for a median of 20 days (IQR, 12.5–30 days) after transfer to an inpatient unit. During this time, 17 cases (68%) and 7 controls (10%) died (Supplementary Figure 1), confirming the greater severity of disease among those in the ICU. Notably, overall survival was significantly shorter in patients admitted to ICU (median, 21 days) than to a medical ward [median not reached; hazard ratio (HR) = 6.32, 95% CI, 2.61–15.26, ${\text{X}}_{\text{LR}}^{2}=$ 22.31; *P* < 0.0001)]. Strikingly, these results maintained significance in the multivariable analysis (HR = 7.07; 95% CI, 2.04–24.54; *P* = 0.002; Supplementary Table 1).

Laboratory tests at presentation (t^0^) showed that cases had lower lymphocyte counts (*P* = 0.003) and higher neutrophil-to-lymphocyte ratios (NLR, *P* = 0.004) than controls (Table 2). Cases also had lower serum sodium (*P* = 0.004), calcium (*P* < 0.001) and albumin (*P* = 0.015), and higher C-reactive protein (P= 0.002), presepsin (*P* = 0.04), D-dimer (*P* = 0.007), lactate dehydrogenase (LDH, *P* < 0.001), hypersensitive troponin I (*P* = 0.003), and NT-proBNP (*P* = 0.04). These differences were confirmed by repeat testing at t^1^, which also showed higher neutrophil counts (*P* < 0.001) and urea-to-creatinine ratios (UCR, *P* < 0.001) in cases (Table 2). In addition, at t^1^ cases had higher serum levels of interleukin-6 (not tested at t^0^; *P* = 0.03).

**Table 2:** Laboratory test results for cases (ICU) and controls (non-ICU), at presentation (t^0^) and admission (t^1^).

	**t**^****0****^			**t**^****1****^			
**Variable**	**ICU (*n* = 25)**	**Non-ICU (*n* = 70)**	***P^a^***	**ICU (*n* = 25)**	**Non-ICU (*n*=70)**	***P^a^***	**Reference values**
Red blood cell count, ×10^12^/L	4.68 (4.27–5.07)	4.71 (4.26–5.08)	0.98	4.1 (3.9–4.5)	4.3 (4.0–4.6)	0.15	3.85–5.16
Hemoglobin, g/dL	13.0 (11.4–14.2)	13.8 (12.3–14.7)	0.35	11.5 (9.9–12.7)	12.7 (11.7–13.7)	0.04	12.0–15.0
White blood cell count, ×10^6^/L	6,220 (4,870–7,610)	5,570 (4,520–7,942)	0.57	7,150 (5,710–10,560)	5,965 (4,500–7,490)	0.01	3,900–11,700
Neutrophil count, ×10^6^/L	5,103 (3,940–6,716)	3,942 (3,204–6,269)	0.17	6,328 (4,900–9,409)	3,751 (2,901–5,628)	<0.001	1,550–8,740
Lymphocyte count, ×10^6^/L	654.0 (595.0–936.1)	994.5 (691.7–1,255.7)	0.003	484.8 (416.5–582.1)	1,058.1 (778.8–1,430.2)	<0.001	820–6,200
Eosinophil count, ×10^6^/L	12.4 (7.1–19.5)	10.5 (6.6–20.2)	0.77	12.8 (6.9–27.5)	53.5 (12.0–107.2)	0.001	20–850
Basophil count, ×10^6^/L	23.7 (11.1–43.1)	27.6 (12.1–159.3)	0.24	21.3 (12.7–48.0)	29.2 (13.4–51.0)	0.68	8–120
Monocyte count, ×10^6^/L	304.8 (266.7–499.4)	359.8 (271.3–508.1)	0.42	314.1 (194.0–390.5)	443.5 (316.3–553.1)	0.009	100–770
Platelet count, ×10^9^/L	155.0 (121.0–226.0)	184.5 (149.2–219.8)	0.46	228.0 (158.0–337.0)	247.0 (183.0–308.0)	0.54	172–440
Neutrophil-to-lymphocyte ratio	7.0 (4.9–11.8)	4.6 (3.0–6.6)	0.004	13.7 (8.1–18.2)	3.5 (2.4–6.0)	<0.001	–
Platelet-to-lymphocyte ratio	0.26 (0.17–0.33)	0.2 (0.15–0.27)	0.08	0.39 (0.27–0.69)	0.23 (0.16–0.31)	<0.001	–
Plasma glucose, mg/dL	97.0 (95.0–122.0)	105.0 (90.0–126.0)	0.94	124.0 (94.0–167.0)	90.5 (75.5–105.8)	<0.001	<100.0
Prothrombin time INR	1.08 (1.04–1.12)	1.06 (1.02–1.10)	0.08	1.10 (1.06–1.17)	1.07 (1.03–1.12)	0.12	<1.2
Activated partial thromboplastin time ratio	1.13 (1.08–1.22)	1.11 (1.04–1.19)	0.51	1.13 (1.04–1.28)	1.11 (1.07–1.19)	0.48	<1.2
Fibrinogen, mg/dL	461.0 (372.8–501.2)	422.0 (378.2–479.2)	0.49	462.0 (435.0–566.0)	441.0 (364.0–518.0)	0.07	200.0–400.0
Creatinine, mg/dL	0.96 (0.79–1.47)	0.91 (0.74–1.12)	0.51	0.78 (0.64–1.00)	0.88 (0.71–1.09)	0.22	0.51–0.95
Estimated glomerular filtration rate, mL/min	73.0 (54.0–87.0)	85.0 (66.8–98.8)	0.20	93.0 (64.0–99.0)	84.5 (72.0–99.8)	0.71	>90.0
Urea, mg/dL	45.0 (30.0–56.0)	34.0 (29.0–41.8)	0.11	40.0 (35.0–67.0)	31.5 (23.0–41.5)	0.004	15.0–38.0
Urea-to-creatinine ratio	43.0 (34.0–51.8)	37.9 (30.3–46.7)	0.14	56.3 (45.2–83.4)	33.8 (25.0–42.3)	<0.001	–
Serum sodium, mmol/L	135.0 (132.0–137.0)	137.0 (135.0–140.0)	0.004	139.0 (138.0–142.0)	139.0 (136.2–141.0)	0.87	136–145
Serum potassium, mmol/L	4.0 (3.7–4.2)	3.9 (3.7–4.3)	0.92	3.9 (3.6–4.2)	4.0 (3.7–4.2)	0.72	3.5–5.0
Serum total calcium, mg/dL	7.9 (7.7–8.2)	8.3 (8.0–8.7)	<0.001	7.6 (7.2–7.8)	8.2 (8.0–8.5)	<0.001	8.5–10.1
Serum albumin-corrected calcium, mg/dL	8.7 (8.6–8.8)	8.9 (8.7–9.1)	0.04	8.8 (8.7–9.2)	9.1 (8.8–9.5)	0.04	8.5–10.1
Albumin, g/dL	3.05 (2.68–3.23)	3.20 (2.92–3.50)	0.015	2.2 (2.0–2.5)	2.8 (2.6–3.2)	<0.001	3.4–5.0
Total plasma protein, g/dL	6.4 (6.33–6.9)	6.6 (6.4–7.2)	0.36	6.0 (5.6–6.3)	6.5 (6.2–6.7)	<0.001	6.4–8.2
Total bilirubin, mg/dL	0.6 (0.5–0.7)	0.5 (0.4–0.6)	0.20	0.6 (0.4–0.9)	0.7 (0.4–0.9)	0.80	0.2–1.0
Direct bilirubin, mg/dL	0.24 (0.18–0.33)	0.19 (0.15–0.24)	0.02	0.28 (0.23–0.47)	0.23 (0.18–0.42)	0.09	0.0–0.2
Indirect bilirubin, mg/dL	0.33 (0.27–0.43)	0.32 (0.25–0.42)	0.58	0.28 (0.17–0.46)	0.36 (0.22–0.62)	0.05	0.0–0.75
Aspartate aminotransferase, U/L	48.5 (42.7–62.8)	36.0 (30.0–60.0)	0.04	50.0 (32.0–65.8)	33.0 (26.3–52.3)	0.02	15.0–37.0
Alanine aminotransferase, U/L	33.0 (27.0–39.0)	33.0 (25.0–44.8)	0.79	29.0 (23.8–40.8)	33.0 (21.3–51.0)	0.52	12.0–78.0
Gamma-glutamyltransferase, U/L	39.0 (28.0–70.0)	46.0 (28.0–69.0)	0.94	39.5 (26.5–83.5)	40.0 (27.0–65.0)	0.88	5.0–55.0
C-reactive protein, mg/L	118.0 (75.8–148.0)	47.8 (26.4–96.0)	0.002	145.0 (107.0–171.2)	49.8 (22.7–108.8)	<0.001	<2.9
Presepsin, pg/mL	531.0 (433.5–914.8)	424.0 (313.0–706.0)	0.04	666.0 (418.5–779.0)	299.0 (209.0–520.0)	0.002	20.0–200.0
D-dimer, μg/L	1,276.0 (718.5–2,816.5)	641.0 (463.5–1,124.5)	0.007	1,936.0 (1,545.0–4,342.0)	635.0 (417.5–1,195.0)	<0.001	<500.0
Lactate dehydrogenase, U/L	410.0 (338.3, 467.0)	281.0 (213.8, 349.8)	<0.001	409.0 (336.0–454.0)	246.5 (204.5–309.8)	<0.001	84.0–246.0
Hypersensitive troponin I, pg/mL	23.9 (15.7–48.6)	10.7 (6.1–26.2)	0.003	38.6 (19.5–56.2)	16.9 (8.8–45.1)	0.16	<74.9
NT-proBNP, pg/mL	342.0 (82.0–1,109.0)	85.0 (44.0–308.0)	0.04	603.0 (368.0–1,770.0)	203.0 (86.8–985.5)	<0.001	0.0–166.0
Interleukin-6, pg/mL	–	–	–	114.0 (36.1–294.5)	33.6 (18.1–105.4)	0.03	0.0–7.0

a *Wilcoxon's rank-sum test*.

*Data are expressed as median and interquartile range*.

We next examined the changes in laboratory test results between presentation (t^0^) and admission (t^1^) (Table 3). During this short interval, neutrophil counts increased in cases but decreased in controls (*P* = 0.006), lymphocyte counts decreased in cases but increased in controls (*P* < 0.001), and eosinophil counts increased only in controls. NLR and UCR values increased in cases but did not change in controls (*P* < 0.001). Serum albumin dropped in both cases and controls, but the change was more severe in cases (*P* = 0.004), and D-dimer increased in cases but did not change in controls (*P* = 0.004).

**Table 3:** Changes in laboratory test results from t^0^ to t^1^.

**Variable (Δ = t^**1**^-t^**0**^)**	**ICU (*n* = 25)**	**Non-ICU (*n* = 70)**	***P^a^***
Red blood cell count, ×10^12^/L	−0.57 (−0.69, −0.25)	−0.36 (−0.65, −0.12)	0.19
Hemoglobin, g/dL	−1.40 (−2.10, −0.80)	−1.20 (−2.00, −0.30)	0.21
White blood cell count, ×10^6^/L	790.00 (−340.00, 2,120.00)	−55.00 (−1,407.50, 1,295.00)	0.10
Neutrophil count, ×10^6^/L	934.17 (136.98, 2,146.06)	−295.13 (−1,397.49, 876.87)	0.006
Lymphocyte count, ×10^6^/L	−184.36 (−321.84, −54.47)	108.56 (−121.70, 354.93)	<0.001
Eosinophil count, ×10^6^/L	0.91 (−12.22, 15.17)	20.87 (2.66, 76.78)	<0.001
Basophil count, ×10^6^/L	−2.51 (−19.80, 9.44)	0.02 (−110.68, 24.91)	0.94
Monocyte count, ×10^6^/L	−75.08 (−165.18, 101.81)	90.31 (−58.32, 166.59)	0.008
Platelet count, ×10^9^/L	51.00 (2.00, 127.00)	52.00 (12.00, 101.50)	0.71
Neutrophil-to-lymphocyte ratio	6.09 (2.53, 10.62)	−0.63 (−2.48, 0.44)	<0.001
Platelet-to-lymphocyte ratio	0.21 (0.04, 0.42)	0.03 (−0.03, 0.08)	<0.001
Plasma glucose, mg/dL	15.00 (−9.00, 32.00)	−15.00 (−37.00, 0.00)	0.001
Prothrombin time INR	0.02 (−0.03, 0.08)	0.00 (−0.03, 0.04)	0.57
Activated partial thromboplastin time ratio	0.04 (−0.08, 0.13)	−0.01 (−0.06, 0.08)	0.51
Fibrinogen, mg/dL	78.50 (−100.25, 121.50)	−0.50 (−70.50, 67.75)	0.23
Creatinine, mg/dL	−0.19 (−0.36, −0.04)	−0.03 (−0.11, 0.09)	<0.001
Estimated glomerular filtration rate, mL/min	13.00 (2.00, 24.00)	1.50 (−5.00, 7.75)	0.001
Urea, mg/dL	5.00 (−4.25, 10.50)	−3.00 (−8.00, 8.00)	0.18
Urea-to-creatinine ratio	20.43 (2.91, 29.41)	−1.89 (−9.92, 7.38)	<0.001
Serum sodium, mmol/L	5.00 (2.00, 7.00)	2.00 (−0.75, 5.00)	0.008
Serum potassium, mmol/L	−0.05 (−0.42, 0.30)	0.00 (−0.40, 0.40)	0.46
Serum total calcium, mg/dL	−0.35 (−0.70, −0.20)	−0.20 (−0.50, 0.20)	0.034
Serum albumin-corrected calcium, mg/dL	0.20 (−0.05, 0.40)	0.10 (−0.10, 0.50)	0.68
Albumin, g/dL	−0.70 (−0.90, −0.55)	−0.40 (−0.60, −0.30)	<0.001
Total plasma protein, g/dL	−0.40 (−0.60, −0.20)	−0.30 (−0.85, 0.10)	0.65
Total bilirubin, mg/dL	0.02 (−0.26, 0.33)	0.10 (−0.08, 0.48)	0.11
Direct bilirubin, mg/dL	0.04 (−0.03, 0.18)	0.04 (−0.02, 0.11)	0.88
Indirect bilirubin, mg/dL	−0.01 (−0.21, 0.08)	0.07 (−0.05, 0.34)	0.01
Aspartate aminotransferase, U/L	−2.00 (−21.75, 11.75)	−4.00 (−14.00, 5.00)	0.76
Alanine aminotransferase, U/L	−2.50 (−8.50, 2.50)	−3.00 (−11.00, 10.75)	0.98
Gamma-glutamyltransferase, U/L	0.00 (−6.50, 5.25)	−3.00 (−9.00, 2.00)	0.55
C-reactive protein, mg/L	30.50 (−33.83, 97.03)	1.10 (−24.70, 33.50)	0.12
Presepsin, pg/mL	84.00 (−160.75, 365.50)	−28.00 (−90.50, 20.50)	0.15
D-dimer, μg/L	733.00 (0.00, 3,267.00)	−46.50 (−198.50, 154.50)	0.004
Lactate dehydrogenase, U/L	35.00 (−76.25, 110.50)	−29.50 (−69.75, 18.00)	0.28
Hypersensitive troponin I, pg/mL	3.70 (−1.90, 22.30)	1.60 (−0.70, 7.10)	0.61
NT-proBNP, pg/mL	174.00 (−25.00, 548.50)	22.50 (−20.27, 117.50)	0.25

a *Wilcoxon's rank-sum test*.

*Data are expressed as median and interquartile range*.

**Figure 1:**
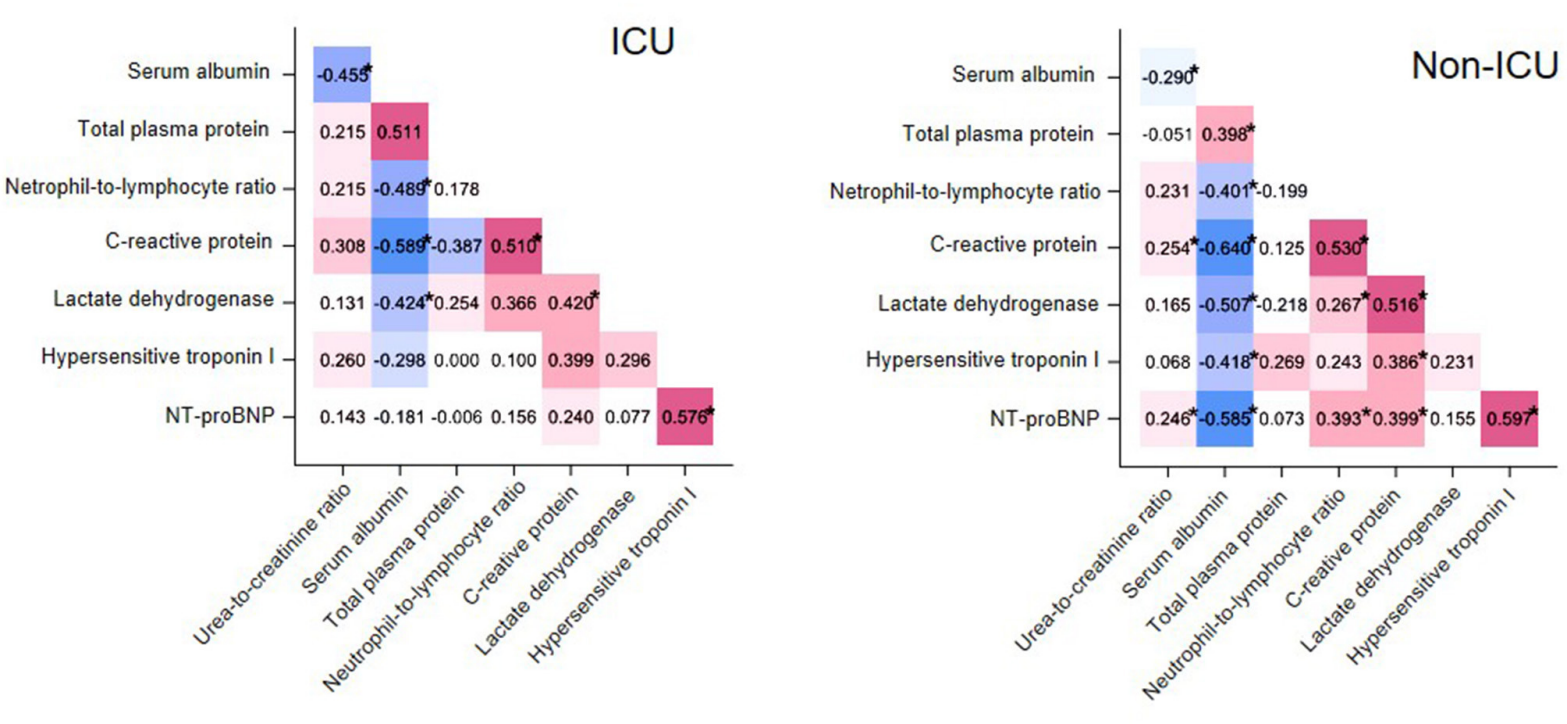
Matrix heatmap representing correlations between laboratory test values in cases (ICU patients, **left**) and controls (non-ICU patients, **right**). Correlations with Spearman's rho < 0 and rho > 0 are colored blue and red, respectively, while the saturation of the color reflects the magnitude of r. *P < 0.05.

To determine if the examined variables had more interaction among themselves than what would be expected from a random set, we examined correlations among selected laboratory test results at t^0^ in cases and controls separately (Figure 1). Specifically, we selected those variables with well-established pathophysiological connections and known alterations in other severe coronavirus-induced diseases. In both groups, NLR correlated negatively with albumin (cases, rho = −0.489, *P* < 0.05; controls, rho = −0.401, *P* < 0.05) and positively with C-reactive protein (cases, rho = 0.510, *P* < 0.05; controls, rho = 0.530, *P* < 0.05). UCR correlated negatively with serum albumin (cases, rho = −0.455, *P* = 0.02; controls: rho = −0.290, *P* = 0.01). A positive correlation also emerged between two critical cardiac parameters, NT-proBNP and hypersensitive troponin I (hsTnI, cases, rho = 0.576, *P* < 0.05; controls, rho = 0.597, *P* < 0.05) in both groups.

**Figure 2:**
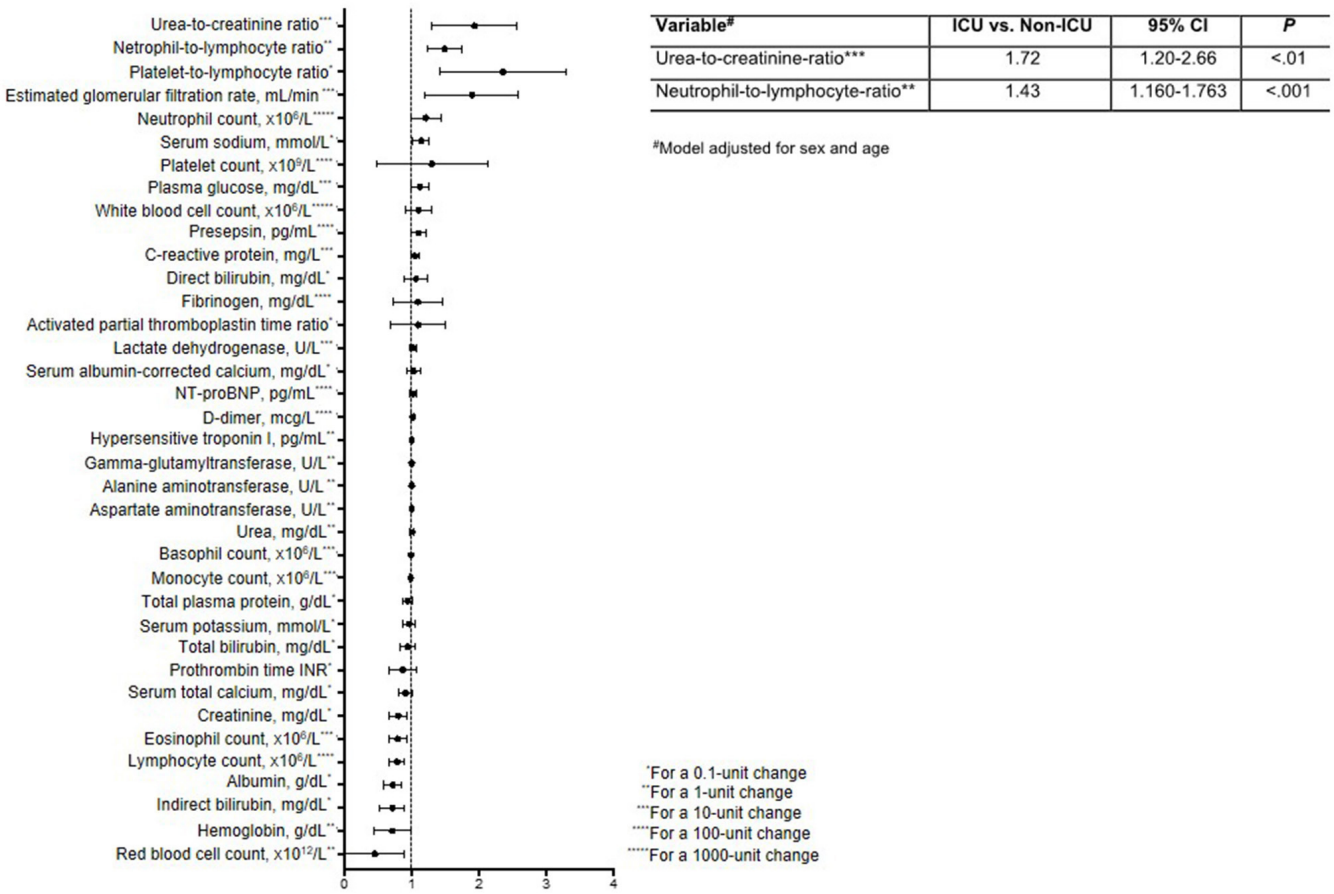
Forest plot reporting odds ratios and 95% confidence intervals for the impact of a short-term change (Δ, t^1^-t^0^) in laboratory variables on the need for ICU admission. The table insert reports the multivariable logistic regression model for predicting ICU status, adjusted for sex and age.

Finally, to identify clinical variables whose short-term change (Δ, t^1^-t^0^) is predictive of ICU admission, we first calculated crude ORs (Figure 2, Supplementary Table 2). This analysis identified six variables with OR > 1 (i.e., cases were more likely to have these variables increase than controls) and seven variables with OR < 1 (i.e., cases were less likely to have these variables increase). These variables were then tested in multivariable logistic regression, which showed that the probability of being admitted to ICU was higher in patients with large increases in UCR (ΔUCR for 10-unit change, OR = 1.72; 95% CI, 1.20–2.66; *P* < 0.01) and NLR (ΔNLR for 1-unit change, OR = 1.430; 95% CI, 1.160–1.763, *P* < 0.001) in the short term. The Hosmer-Lemeshow goodness-of-fit test indicated that the model appropriately described the data [X^2^(8) = 5.29, *P* = 0.73].

## Discussion

This study identified two critical short-term changes in laboratory variables that are sufficient to sketch the identikit of a COVID-19 patient deserving close clinical monitoring and high-intensity care. These changes regard the neutrophil-to-lymphocyte ratio (NLR) and urea-to-creatinine ratio (UCR), which both increased in cases requiring ICU admission but did not change significantly in controls treated in a nonintensive medical ward. The changes in these two variables are statistically robust enough to explain, independently, patient assignment or not to the ICU.

An increase in NLR is likely to parallel the strength of the immune-inflammatory response and cytokine storm, two phenomena that have been widely described in COVID-19 (14, 15). This increase can depend on neutrophilia, lymphopenia or both. Neutrophilia might be due to a virus-related cytokine storm, coagulation activation, hypoxia or shock (12, 16). Interestingly, an accumulation of inflammatory neutrophils and monocyte-macrophages was promoted, in a mouse model of SARS, by the rapid kinetics of virus replication and the subsequent delay in interferon type I signaling (17). Lymphopenia is believed to be determined by tissue sequestration, cell destruction in peripheral blood, or bone marrow failure singly or together (18, 19), as already demonstrated in MERS-CoV and SARS-CoV infections (20, 21). Lymphocyte sequestration, in particular, seems to play the major role and has been shown to occur in the lungs, gastrointestinal tract, and lymphoid tissues of COVID-19 patients (22, 23).

An increase in UCR is likely to reflect the acute catabolic state (24) and renal injury (25) that characterize the critical phase of COVID-19. The dynamic UCR profiles (urea increase, and creatinine decrease or stability) of our patients seem to contradict recent evidence that high serum creatinine concentration correlates with catastrophic COVID-19 outcome (acute kidney injury or death) (26). Our findings can be explained by the simultaneous decrease in serum albumin; decreases in albumin are associated with muscle catabolism and persistent critical illness (27, 28) and are found in other chronic disease states (29, 30).

It should be noted that the use of corticosteroids during hospitalization may also account for a decrease in lymphocytes, as already observed in SARS patients (31), and that fluid restriction strategies in the management of COVID-19 patients may contribute to the UCR elevation. Although we cannot entirely rule out these confounding variables when interpreting our results, we also cannot overstate that the use of corticosteroids in our study population was negligible and that no restrictive fluid protocols were followed, given the absence of consensus recommendations.

Our study has some other limitations. First, the sample size might not have been adequate to assess risk factors for poor clinical outcome. Second, due to the retrospective study design, we might have underestimated some laboratory and clinical findings predicting in-hospital mortality. Statistically powered clinical trials are therefore needed to corroborate our findings. Finally, both NLR and UCR are not disease-specific and need to be supported by clinical judgment.

The predictive significance of NLR for COVID-19 severity and mortality was evaluated in several studies whose findings have been used in five meta-analyses (32–36), all finding that NLR was higher in patients with than without severe disease. So far, only one single-center study reported that UCR, alone or in combination with NLR, was an independent predictor of COVID-19 severity (37). In all studies, however, only single time points were considered. Our study extends these findings by showing that the magnitude of short-term variations in these two measures are also prognostic.

The construction and validation of new parameters to aid in severity assessment of COVID-19 patients are crucial. This, however, is complicated by the heterogeneous host response to infection, as well as the delayed onset of severe manifestations. Our study proposes short-time variations in NLR and UCR as novel indicators of disease severity that can contribute to the construction of a standardized set of criteria supporting the early identification of severe COVID-19 cases that require intensive care admission. These fast, affordable markers of disease severity should be evaluated before the onset of severe manifestations. If confirmed prospectively, they should facilitate the proper allocation of patients and the better use of resources for a disease that is putting enormous pressure on health systems all around the world.

## Data Availability Statement

The raw data supporting the conclusions of this article will be made available by the authors, without undue reservation.

## Ethics Statement

The studies involving human participants were reviewed and approved by Comitato Etico Indipendente Azienda Ospedaliero-Universitaria Consorziale Policlinico - Bari (n. 6357). Written informed consent for participation was not required for this study in accordance with the national legislation and the institutional requirements.

## Author Contributions

AGS, NSu, PB, and VR contributed to study conception and design. GL, FP, RB, GI, BF, DB, MC, VC, SC, and PL contributed to data collection. PB, MP, NSu, AS, RR, VP, GM, AV, AGS, NSi, and VR contributed to data analysis. AGS, NSu, AV, and VR contributed to drafting and editing the manuscript. All authors contributed to the article and approved the submitted version.

## Conflict of Interest

The authors declare that the research was conducted in the absence of any commercial or financial relationships that could be construed as a potential conflict of interest.

## Supplementary Material

Supplementary Figure 1 Kaplan-Meier estimates of overall survival (OS) stratified for ICU admission status. ${\text{X}}_{\text{LR}}^{2}$, chi-squared log-rank test. NR, not reached.

Supplementary Table 1 Cox proportional hazards model for overall survival.

Supplementary Table 2 Odds ratios (ORs) and 95% confidence intervals for ICU admission, according to changes (t^1^-t^0^) in laboratory variables.
