# Supplementary figures and images for: Short-Term Variations in Neutrophil-to-Lymphocyte and Urea-to-Creatinine Ratios Anticipate Intensive Care Unit Admission of COVID-19 Patients in the Emergency Department
Source: Front Med (Lausanne). 2021 Jan 20;7:625176. doi: 10.3389/fmed.2020.625176 (PMC7854700; doi:10.3389/fmed.2020.625176)

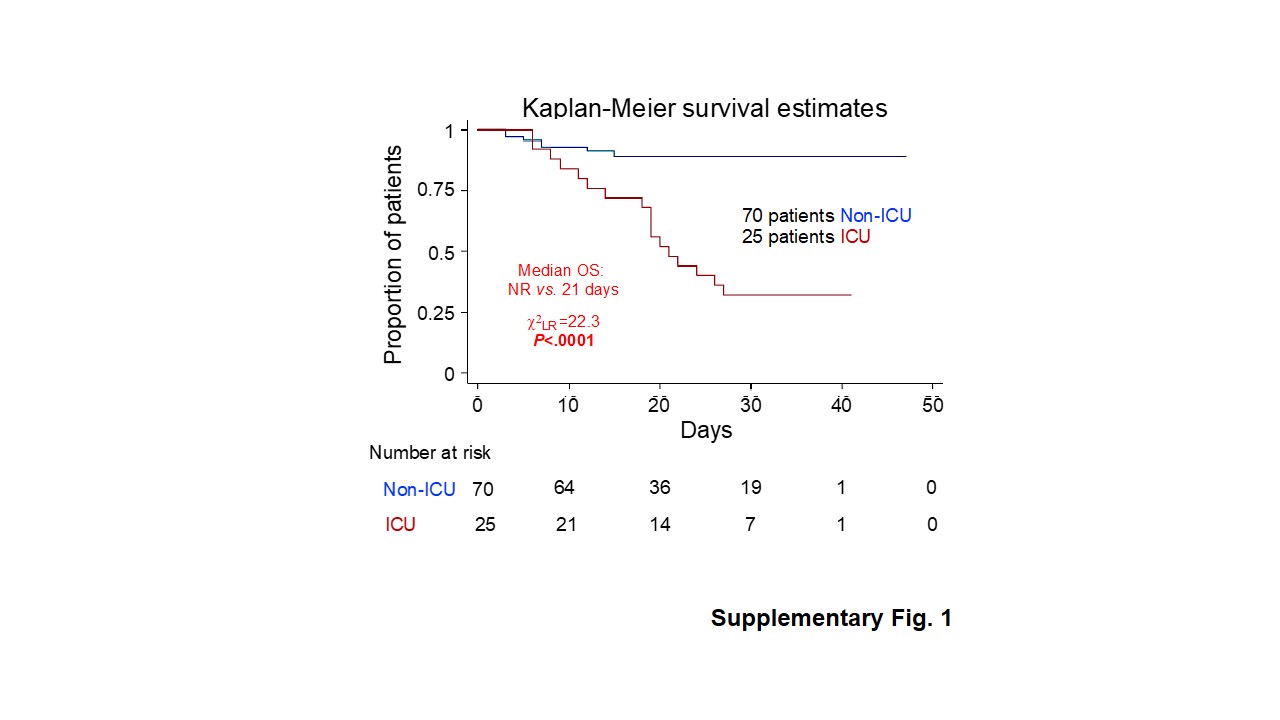

Supplement: Supplementary Figure 1 — Kaplan-Meier estimates of overall survival (OS) stratified for ICU admission status. XLR2, chi-squared log-rank test. NR, not reached. [file Image_1.JPEG]
