# Supplementary material for: Short-Term Variations in Neutrophil-to-Lymphocyte and Urea-to-Creatinine Ratios Anticipate Intensive Care Unit Admission of COVID-19 Patients in the Emergency Department
Source: Front Med (Lausanne). 2021 Jan 20;7:625176. doi: 10.3389/fmed.2020.625176 (PMC7854700; doi:10.3389/fmed.2020.625176)
Supplement: Supplementary Table 1 — Cox proportional hazards model for overall survival. [file Table_1.DOCX]

**Supplementary Table 1.** Cox proportional hazards model for overall survival

| **Variable** | **Univariate analysis** | | **Multivariate analysis** | |
| --- | --- | --- | --- | --- |
|  | **HR (95% CI)** | ***P*** | **HR (95% CI)** | ***P*** |
| ICU admission | 6.32 (2.61-15.26) | <.001 | 7.07 (2.04-24.54) | .002 |
| Age, y^a^ | 1.06 (1.03-1.10) | <.001 | 1.08 (1.02-1.13) | .002 |
| Male gender | 1.51 (0.62-3.68) | .36 | 1∙13 (0.34-3.73) | .83 |
| Current smoking habit | 7.21 (0.92-56.62) | .06 | - | - |
| Systolic arterial pressure, mmHg^a^ | 1.01 (0.98-1.04) | .33 | - | - |
| Diastolic arterial pressure, mmHg^a^ | 0.99 (0.94-1.03) | .77 | - | - |
| Heart rate, bpm^a^ | 0.97 (0.95-1.00) | .11 | - | - |
| Body temperature, °C^a^ | 0.60 (0.33-1.07) | .08 | - | - |
| Peripheral capillary oxygen saturation, %^a^ | 0.94 (0.85-1.05) | .32 | - | - |
| Fraction of inspired oxygen, %^a^ | 1.02 (1.00-1.03) | .003 | - | - |
| Dyspnea | 2.22 (0.87-5.65) | .09 | - | - |
| Noninvasive ventilation in ED | 5.03 (2.08-12.19) | <.001 | 1.30 (0.39-4.34) | .67 |
| Hypertension | 3.64 (1.33-9.94) | .01 | 1.18 (0.34-4.07) | .79 |
| Obesity | 4.58 (1.88-11.16) | .001 | - | - |
| Chronic heart failure | 4.38 (1.85-10.39) | .001 | 2.49 (0.86-7.16) | 0.09 |
| COPD | 2.06 (0.76-5.58) | .15 | - | - |
| Type 2 diabetes mellitus | 2.07 (0.81-5.26) | .13 | - | - |
| Allergy | 0.87 (0.20-3.75) | .86 | - | - |
| CNS disease | 2.72 (1.01-7.36) | .05 | - | - |
| Atrial fibrillation | 2∙72 (0∙80-9∙19) | ∙11 | - | - |
| Chronic kidney disease (≥III KDOQI) | 0.42 (0.05-3.13) | .40 | - | - |
| Autoimmune disease | 1.36 (0.18-10.13) | .76 | - | - |
| Antiplatelet drugs | 2.56 (0.99-6.63) | .052 | - | - |
| ACE inhibitors or ARB | 2.81 (1.01-7.73) | .05 | - | - |
| Other antihypertensive drugs | 3.21 (1.14-9.06) | .03 | - | - |
| Inhalants | 1.55 (0.20-11.73) | .67 | - | - |
| Warfarin or DOAC | 2.73 (0.79-9.48) | .11 | - | - |
| Systemic corticosteroids | 25.46 (4.57-141.79) | <.001 | - | - |

^a^ For a one-unit increase.

Abbreviations HR (95% C.I.), hazard ratio and 95% confidence interval; ACE, angiotensin-converting enzyme; ARB, angiotensin receptor blockers; CNS, central nervous system; COPD, chronic obstructive pulmonary disease; DOAC, direct oral anticoagulants; ED, emergency department; KDOQI, Kidney Disease Outcomes Quality Initiative
