# Supplementary material for: Short-Term Variations in Neutrophil-to-Lymphocyte and Urea-to-Creatinine Ratios Anticipate Intensive Care Unit Admission of COVID-19 Patients in the Emergency Department
Source: Front Med (Lausanne). 2021 Jan 20;7:625176. doi: 10.3389/fmed.2020.625176 (PMC7854700; doi:10.3389/fmed.2020.625176)
Supplement: Supplementary Table 2 — Odds ratios (ORs) and 95% confidence intervals for ICU admission, according to changes (t1-t0) in laboratory variables. [file Table_2.DOCX]

**Supplementary Table 2.**

Odds ratios (ORs) and 95% confidence intervals for ICU admission, according to changes (t^1^-t^0^) in laboratory variables

| **Variable ( Δ= t^1^-t^0^)** | **OR** | **95% CI** |
| --- | --- | --- |
| Red blood cell count, x10^12^/L^a^ | 0.32 | 0.10-0.94 |
| Hemoglobin, g/dL^a^ | 0.68 | 0.45-1.00 |
| White blood cell count, x10^6^/L^b^ | 1.09 | 0.92-1.30 |
| Neutrophil count, x10^6^/L^b^ | 1.19 | 1.00-1.45 |
| Lymphocyte count, x10^6^/L^c^ | 0.78 | 0.67-0.90 |
| Eosinophil count, x10^6^/L^d^ | 0.80 | 0.66-0.92 |
| Basophil count, x10^6^/L^d^ | 0.99 | 0.98-1.00 |
| Monocyte count, x10^6^/L^d^ | 0.98 | 0.97-1.00 |
| Platelet count, x10^9^/L^c^ | 1.13 | 0.57-2.20 |
| Neutrophil-to-lymphocyte ratio^a^ | 1.45 | 1.25-1.76 |
| Platelet-to-lymphocyte ratio^a^ | 2.17 | 1.53-3.37 |
| Plasma glucose, mg/dL^d^ | 1.11 | 1.00-1.26 |
| Prothrombin time INR ^e^ | 0.93 | 0.64-1.02 |
| Activated partial thromboplastin time ratio^e^ | 1.04 | 0.72-1.52 |
| Fibrinogen, mg/dL^c^ | 1.04 | 0.76-1.48 |
| Creatinine, mg/dL^e^ | 0.80 | 0.67-0.93 |
| Estimated glomerular filtration rate, mL/min^e^ | 1.78 | 1.27-2.64 |
| Urea, mg/dL^a^ | 1.00 | 0.98-1.03 |
| Urea-to-creatinine ratio^d^ | 1.83 | 1.36-2.61 |
| Serum sodium, mmol/L^a^ | 1.13 | 1.02-1.27 |
| Serum potassium, mmol/L^e^ | 0.96 | 0.88-1.05 |
| Serum total calcium, mg/dL^e^ | 0.91 | 0.81-1.00 |
| Serum albumin-corrected calcium, mg/dL^e^ | 1.02 | 0.93-1.13 |
| Albumin, g/dL^e^ | 0.72 | 0.58-0.85 |
| Total plasma protein, g/dL^e^ | 0.96 | 0.85-1.00 |
| Total bilirubin, mg/dL^e^ | 0.94 | 0.83-1.04 |
| Direct bilirubin, mg/dL^e^ | 1.05 | 0.90-1.24 |
| Indirect bilirubin, mg/dL^e^ | 0.71 | 0.53-0.90 |
| Aspartate aminotransferase, U/L^a^ | 1.00 | 0.99-1.01 |
| Alanine aminotransferase, U/L^a^ | 1.00 | 0.99-1.01 |
| Gamma-glutamyltransferase, U/L^a^ | 1.00 | 0.99-1.01 |
| C-reactive protein, mg/L^d^ | 1.05 | 0.98-1.12 |
| Presepsin, pg/mL^c^ | 1.08 | 1.00-1.23 |
| D-dimer, μg/L^c^ | 1.01 | 0.99-1.04 |
| Lactate dehydrogenase, U/L^d^ | 1.02 | 0.98-1.07 |
| Hypersensitive troponin I, pg/mL^a^ | 1.00 | 0.99-1.01 |
| NT-proBNP, pg/mL^c^ | 1.01 | 0.97-1.07 |

^a^ For a 1-unit change; ^b^ For a 1000-unit change; ^c^ For a 100-unit change; ^d^ For a 10-unit change; ^e^ For a 0.1-unit change
